# Progressive Bilateral Sensorineural Hearing Loss and Ataxia Caused by Superficial Siderosis Secondary to Chronic Spontaneous Intracranial Hypotension

**DOI:** 10.7759/cureus.97814

**Published:** 2025-11-25

**Authors:** Jerin G Viju, Parameswaran Krishnan, Anand M, Ashik Anilkumar

**Affiliations:** 1 Neurology, Indo American Hospital, Vaikom, IND; 2 Neuroradiology, Indo American Hospital, Vaikom, IND; 3 Emergency Medicine, Indo American Hospital, Vaikom, IND

**Keywords:** deferiprone, epidural blood patch, hemosiderin deposition, mri imaging, neuro radiology

## Abstract

Superficial siderosis (SS) is an uncommon neurodegenerative disorder resulting from persistent or repeated bleeding into the subarachnoid space, which causes hemosiderin to accumulate along the subpial surfaces of the brain, brainstem, spinal cord, and cranial nerves. Chronic spontaneous intracranial hypotension (SIH) due to a dural tear is an uncommon but treatable condition. We report a 51-year-old man with a seven-year history of progressive bilateral sensorineural hearing loss, disabling gait ataxia, vertigo, tinnitus, and facial numbness. Ten years earlier, he had experienced a prolonged, classical orthostatic headache lasting four to six months, which resolved after unspecified treatment. Magnetic resonance imaging (MRI) of the brain revealed features of SS involving the posterior fossa, and an MRI whole-spine survey with fat-suppressed sampling perfection with application-optimized contrasts (SPACE) sequence identified a cervicodorsal, longitudinally extensive extradural cerebrospinal fluid (CSF) collection. The brain MRI also demonstrated a reduced ponto-mesencephalic angle, consistent with intracranial hypotension. A diagnosis of SS secondary to chronic SIH from a spinal dural tear was made. He underwent an autologous epidural blood patch, followed by a second patch, which resulted in symptomatic stabilization, along with iron chelation and corticosteroid therapy. This case highlights the importance of recognizing SS as a delayed complication of untreated SIH. Timely identification and repair of dural defects can halt progression and potentially reverse neurological deficits.

## Introduction

Superficial siderosis (SS) of the central nervous system is a rare, slowly progressive neurological disorder characterized by hemosiderin deposition along the pial surfaces as a result of chronic or recurrent bleeding into the subarachnoid space [1]. The hemosiderin, derived from the breakdown of hemoglobin, is most sensitively detected using iron-sensitive magnetic resonance imaging (MRI) sequences, such as susceptibility-weighted imaging (SWI) and gradient-recalled echo (GRE) [1].

SS is categorized into the following two main forms based on its anatomical distribution: cortical superficial siderosis (cSS) and infratentorial superficial siderosis (iSS) [2,3]. Among these, iSS is further subdivided into type 1 (classical iSS) and type 2 (secondary iSS) [2,3]. Classical iSS refers to cases in which there is no radiologically confirmed single spontaneous or traumatic intracranial hemorrhage and is typically associated with chronic bleeding sources, such as cranial or spinal dural defects [2,3]. In contrast, secondary iSS is characterized by the presence of a radiologically identifiable single intracranial hemorrhagic event, either spontaneous or traumatic, which is considered the likely cause of the siderosis [2,3].

The classical clinical presentation is a triad of progressive bilateral sensorineural hearing loss, cerebellar ataxia, and pyramidal signs, although not all features are present in every patient [1]. Over time, the condition is often disabling, with hearing loss typically irreversible if left untreated. The most frequent underlying cause is spinal dural pathology, particularly dural tears, which can occur spontaneously or secondary to trauma, degenerative disc disease, or surgical intervention [1].

Spontaneous intracranial hypotension (SIH), typically caused by cerebrospinal fluid (CSF) leaks from dural tears, is a well-recognized cause of orthostatic headaches but is less commonly associated with SS [4,5]. Chronic CSF leakage can lead to venous engorgement and traction on fragile vessels in the posterior fossa, resulting in recurrent microhemorrhages and hemosiderin deposition [4]. We report a patient in whom SS developed years after an episode of classical SIH headache, highlighting a prolonged latency between the initial leak and the onset of irreversible neurological symptoms. Although the association between dural tears, SIH, and superficial siderosis has been increasingly recognized, reports describing a prolonged latency of many years between the initial orthostatic headache and the onset of SS are scarce [4]. The present case contributes to this limited body of literature.

## Case presentation

This case highlights the clinical continuum between SIH and SS. While most patients with SIH present acutely with orthostatic headache and are diagnosed early, delayed recognition may allow years of ongoing occult bleeding, ultimately leading to progressive neurological decline [5,6].

A 51-year-old man presented to the neurology outpatient department with a seven-year history of progressive bilateral hearing loss, more pronounced on the right, accompanied by vertigo, swaying while walking, tinnitus, severe unsteadiness on standing and turning, and facial numbness. The hearing loss had progressed to the point that he had ceased his business activities and avoided social interactions due to communication difficulties.

Ten years earlier, he experienced a disabling orthostatic headache that worsened on sitting or standing and improved on lying down, lasting four to six months before resolving after unspecified treatment. There was no history of trauma, CSF otorrhea, spinal surgery, meningitis, or any acute neurological illness during that period, reducing the likelihood of post-traumatic or post-meningitic causes of hemosiderin deposition.

Neurological examination revealed bilateral sensorineural hearing loss with minimal residual hearing on the left, no nystagmus, and Rinne’s test was positive bilaterally with a non-lateralizing Weber’s test. Absolute bone conduction was reduced bilaterally. The head impulse test was positive bilaterally with catch-up saccades, suggesting bilateral vestibular hypofunction. Dix-Hallpike, half Dix-Hallpike, and supine roll tests were negative, effectively ruling out benign paroxysmal positional vertigo (BPPV). There were no cerebellar limb signs or long tract signs, but gait was broad-based and unsteady with marked postural instability. Sensory examination was normal, excluding peripheral neuropathy as a primary cause of imbalance.

He had type 2 diabetes mellitus (HbA1c: 14.2%) and hypertension; other laboratory parameters were unremarkable, making metabolic or inflammatory neuropathies less likely. Given the chronicity of symptoms, absence of peripheral sensory deficits, combined auditory and vestibular involvement, and a past history of orthostatic headache suggestive of spontaneous intracranial hypotension, a central cause involving the brainstem and cranial nerves was suspected.

Neuroimaging revealed classical infratentorial superficial siderosis, correlating with the patient’s progressive eighth cranial nerve dysfunction, vestibular impairment, and postural instability. MRI brain demonstrated hemosiderin deposition along the posterior fossa structures. A fat-suppressed sampling perfection with application-optimized contrasts (SPACE) sequence MRI of the whole spine identified a longitudinally extensive cervicodorsal extradural CSF collection. Additionally, MRI brain showed partial effacement of the lateral ventricle, downward sagging of the corpus callosum, and a reduced ponto-mesencephalic angle of 44.2° (the normal ponto-mesencephalic angle measures 65°±10°, with values below 50° indicating intracranial hypotension), consistent with intracranial hypotension [4]. These findings established the diagnosis of superficial siderosis secondary to chronic spontaneous intracranial hypotension due to a cervical dural tear.

The patient underwent an autologous epidural blood patch, followed by a second patch two weeks later under image guidance to achieve near-complete sealing of the CSF leak. He was also started on iron chelation therapy and a short course of corticosteroids (with strict glycemic control) to help mitigate oxidative damage from hemosiderin deposition.

MRI and relevant imaging

**Figure 1:**
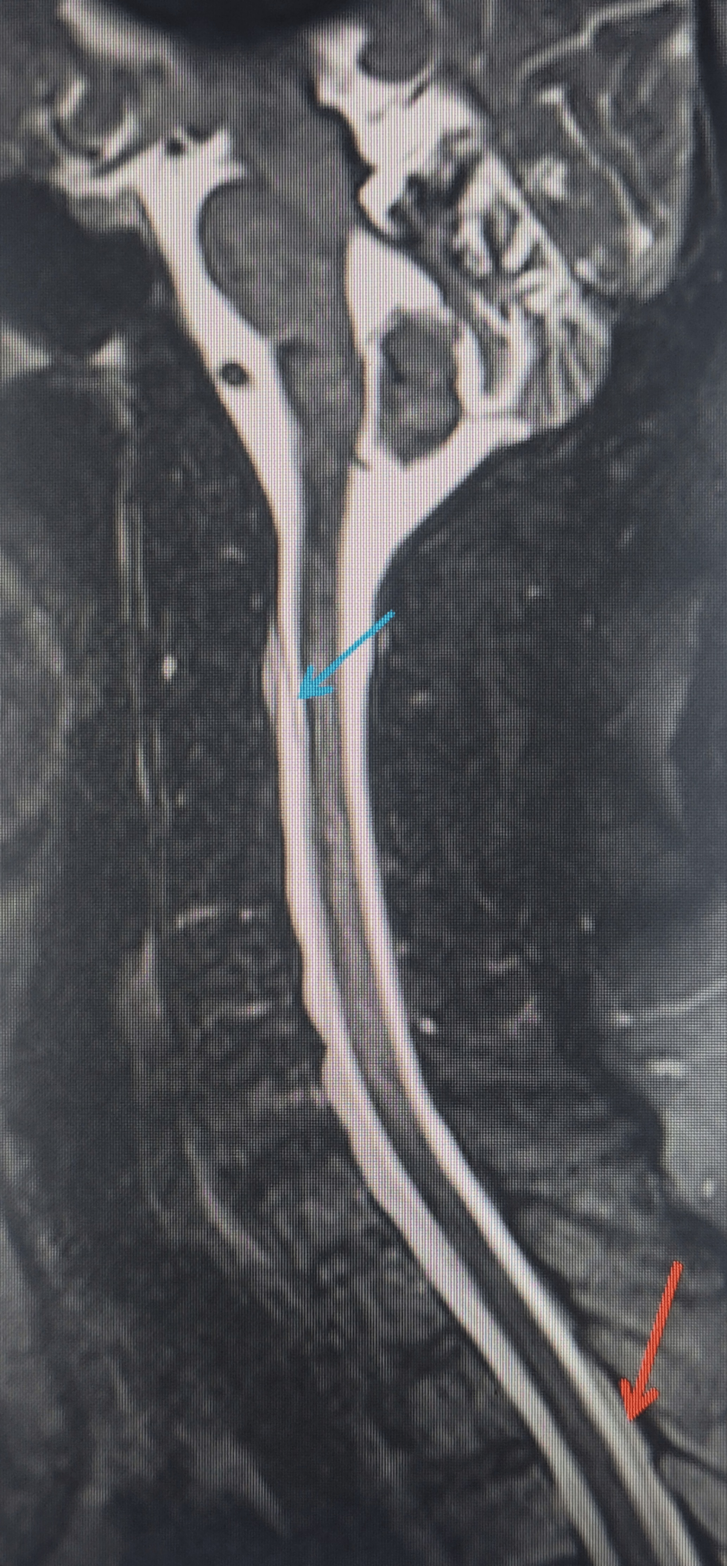
Sagittal T2-weighted fat-suppressed SPACE image of the spine showing ventral and dorsal epidural fluid collections. The image demonstrates hyperintense epidural fluid collections both ventrally (blue arrow) and dorsally (orange arrow), distinctly separated from the subarachnoid space by the intervening dural lining. SPACE: sampling perfection with application-optimized contrasts

**Figure 2:**
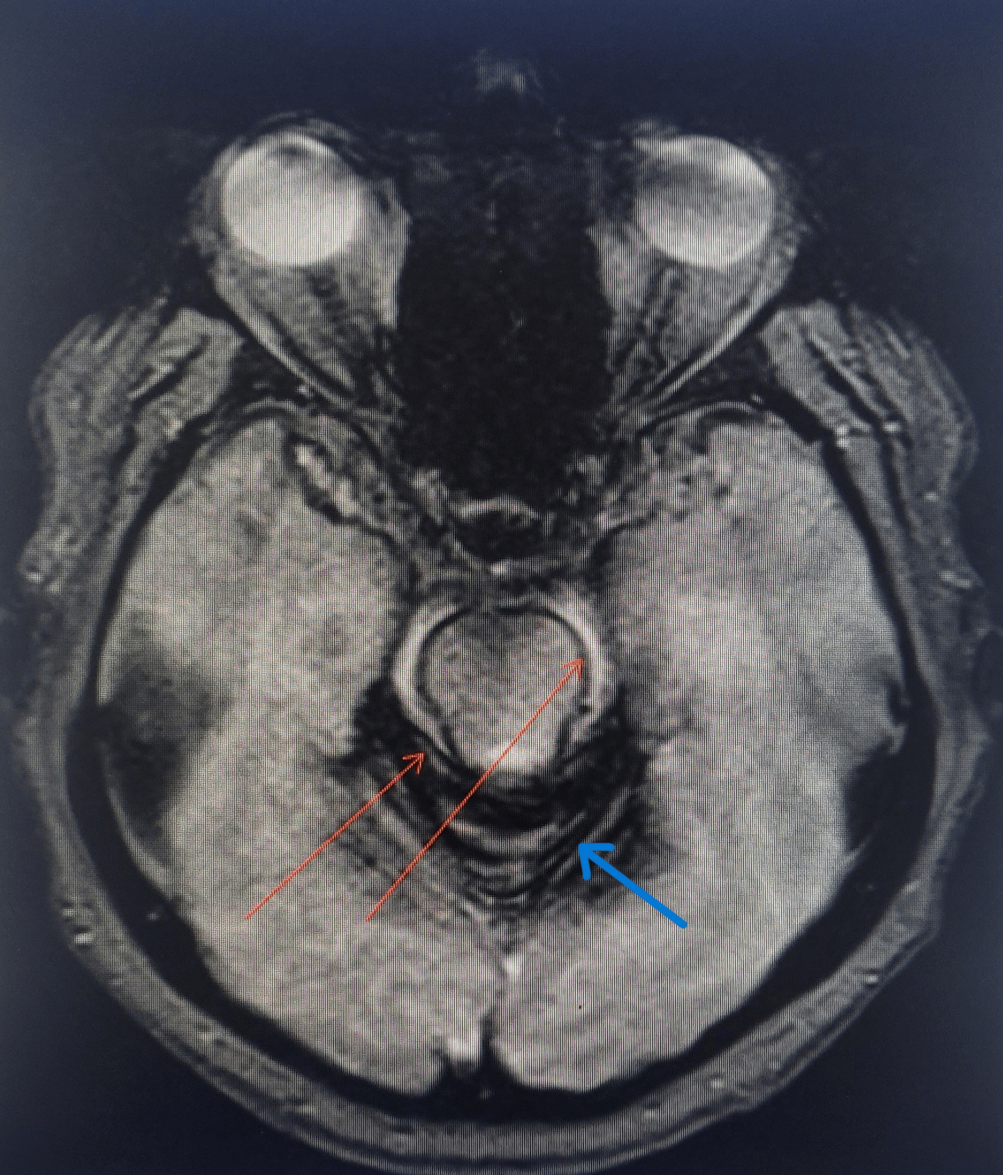
Axial GRE image at the level of pons showing a hypointense siderotic rim along the brainstem and cerebellar folia. The image demonstrates a hypointense siderotic rim along the subarachnoid surfaces of the brainstem (orange arrows) and cerebellar folia (blue arrow). GRE: gradient-recalled echo

Sagittal T2-weighted fat-suppressed SPACE image of the spine shows ventral and dorsal epidural hyperintense collections separated from the subarachnoid space by the intact dura (Figure 1). Axial gradient-recalled echo (GRE) images demonstrate a hypointense siderotic rim along the subarachnoid surfaces of the brainstem and cerebellar folia (Figure 2).

**Figure 3:**
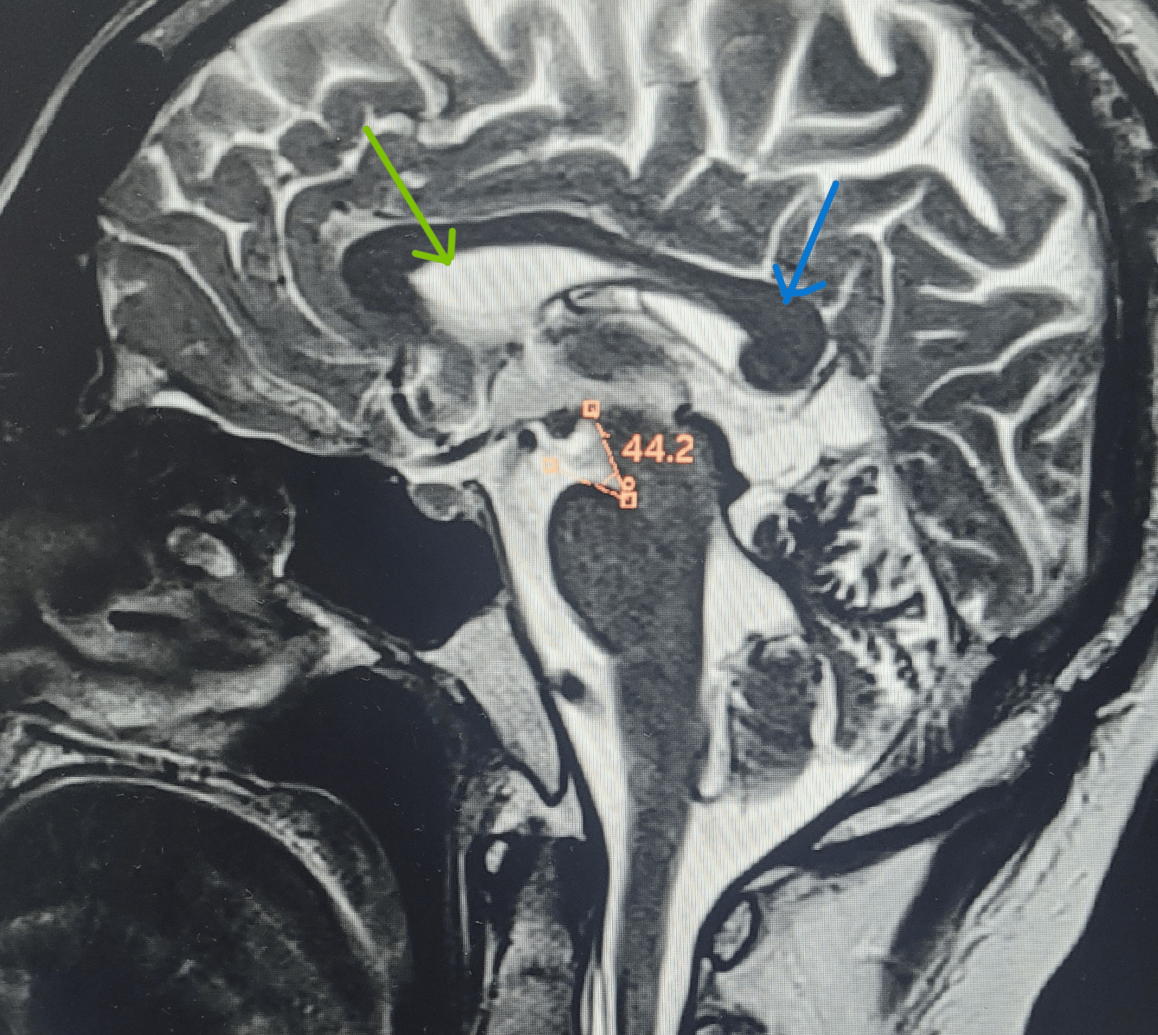
Sagittal T2 image showing brain sagging with reduced ponto-mesencephalic angle. The image demonstrates partial effacement of the lateral ventricle (green arrow), downward sagging of the corpus callosum (blue arrow), and a reduced ponto-mesencephalic angle of 44.2°.

Sagittal T2-weighted image demonstrates partial effacement of the lateral ventricle, downward sagging of the corpus callosum, and a reduced ponto-mesencephalic angle, consistent with brain sagging secondary to intracranial hypotension (Figure 3).

## Discussion

SS is a rare neurodegenerative condition caused by chronic or recurrent bleeding into the subarachnoid space, leading to hemosiderin deposition along the subpial surfaces of the brain, spinal cord, and cranial nerves. The brain’s capacity to synthesize ferritin in response to prolonged exposure to hemoglobin-derived iron plays a key role in its pathogenesis. Accelerated ferritin production within the Bergmann glia of the cerebellum may account for the selective vulnerability of cerebellar tissue. Ferritin initially exerts a neuroprotective effect by sequestering free iron; however, once this buffering capacity is overwhelmed, excess intrathecal iron promotes free radical formation and lipid peroxidation, resulting in neuronal injury. The classical clinical triad comprises progressive sensorineural hearing loss, cerebellar ataxia, and pyramidal signs, though additional manifestations, such as vertigo, tinnitus, and cranial neuropathies, may also occur [1].

In this patient, the antecedent history of prolonged orthostatic headache a decade earlier was highly suggestive of spontaneous intracranial hypotension (SIH), a condition commonly arising from a spinal dural defect. If unrecognized or untreated, SIH can lead to persistent cerebrospinal fluid (CSF) leakage and subsequent bleeding from fragile bridging veins, ultimately causing SS [4,5]. This highlights the importance of considering SIH in patients with classical orthostatic headaches, as timely intervention may prevent irreversible neurological sequelae.

Neuroimaging plays a central role in diagnosis [1]. In our case, brain MRI demonstrated infratentorial hemosiderin deposition characteristic of SS, while whole-spine MRI with fat-suppressed sequences revealed a cervicodorsal extradural CSF collection consistent with a dural tear. Additionally, the reduced ponto-mesencephalic angle on brain imaging provided supportive evidence of intracranial hypotension [7].

The management of SS primarily aims to eliminate or control the source of bleeding. In cases of SIH-related dural defects, targeted epidural blood patching remains the cornerstone of treatment, with repeat interventions often required [4]. In our patient, two autologous epidural blood patches led to symptomatic stabilization. Adjunctive therapy with deferiprone, an iron-chelating agent capable of crossing the blood-brain barrier, was initiated to mitigate hemosiderin deposition. A short course of corticosteroids was also administered to limit inflammation and prevent further neuronal damage. The role of adjunctive therapies, such as iron chelation and corticosteroids, remains under investigation, with mixed evidence regarding long-term benefit [8]. However, given the potential to reduce oxidative damage, these were considered reasonable adjuncts in our patient.

This case underscores the importance of recognizing SS as a delayed but preventable complication of untreated SIH. Early identification and repair of dural defects are crucial to halting disease progression and may preserve neurological function.

## Conclusions

Superficial siderosis is a progressive but often underrecognized consequence of chronic spontaneous intracranial hypotension due to spinal dural defects. This case highlights the importance of carefully eliciting a past history of orthostatic headache in patients presenting with progressive auditory and cerebellar symptoms, as this may provide a crucial diagnostic clue. Early identification and repair of dural leaks, combined with adjunctive therapies, can halt disease progression and potentially restore neurological function. Clinicians should maintain a high index of suspicion for SS in patients with unexplained bilateral sensorineural hearing loss and ataxia, as timely intervention may prevent irreversible disability.
